# Impact of some types of mass gatherings on current suicide risk in an urban population: statistical and negative binominal regression analysis of time series

**DOI:** 10.1186/1471-2458-14-308

**Published:** 2014-04-04

**Authors:** Vasiliy S Usenko, Sergey N Svirin, Yan N Shchekaturov, Eduard D Ponarin

**Affiliations:** 1 Scientific Centre ‘Medical Technologies’, 52A/88 Komsomolskaya str., Dnipropetrovsk 49000, Ukraine; 2 Municipal Hospital # 14’, 12A Fuchika str., Dnipropetrovsk 49044, Ukraine; 3 Dnipropetrovsk Help Centre for Victims of Destructive Cults ‘Dialogue’, p/b 1793, Dnipropetrovsk 49027, Ukraine; 4 Laboratory for Comparative Social Research, Higher School of Economics, rm 313, 3 Tenth Line VO, St. Petersburg 199034, Russia

**Keywords:** Suicide, Suicide attempt, Mass gatherings, Football, Concerts, Religion, New religious movements, Broken-promises effect, Conflict, Crowd behaviour

## Abstract

**Background:**

Many studies have investigated the impact of a wide range of social events on suicide-related behaviour. However, these studies have predominantly examined national events. The aim of this study is to provide a statistical evaluation of the relationship between mass gatherings in some relatively small urban sub-populations and the general suicide rates of a major city.

**Methods:**

The data were gathered in the Ukrainian city of Dnipropetrovsk, with a population of 1 million people, in 2005–2010. Suicide attempts, suicides, and the total amount of suicide-related behaviours were registered daily for each sex. Bivariate and multivariate statistical analysis, including negative binomial regression, were applied to assess the risk of suicide-related behaviour in the city’s general population for 7 days before and after 427 mass gatherings, such as concerts, football games, and non-regular mass events organized by the Orthodox Church and new religious movements.

**Results:**

The bivariate and multivariate statistical analyses found significant changes in some suicide-related behaviour rates in the city’s population after certain kinds of mass gatherings. In particular, we observed an increased relative risk (RR) of male suicide-related behaviour after a home defeat of the local football team (RR = 1.32, p = 0.047; regression coefficient beta = 0.371, p = 0.002), and an increased risk of male suicides (RR = 1.29, p = 0.006; beta =0.255, p = 0.002), male suicide-related behaviour (RR = 1.25, p = 0.019; beta =0.251, p < 0.001), and total suicide-related behaviour (RR = 1.23 p < 0.001; beta =0.187, p < 0.001) after events organized by the new religious movements.

**Conclusions:**

Although football games and mass events organized by new religious movements involved a relatively small part of an urban population (1.6 and 0.3%, respectively), we observed a significant increase of the some suicide-related behaviour rates in the whole population. It is likely that the observed effect on suicide-related behaviour is related to one’s personal presence at the event rather than to its broadcast. Our findings can be explained largely in terms of Gabennesch’s theory of the ‘broken-promises effect’ with regard to intra- and interpersonal conflict and, in terms of crowd behaviour effects.

## Background

The success of any suicide-prevention programme depends on our ability to identify and influence the factors that considerably increase the risk of suicide in the population. This risk is primarily affected by social psychological factors whose effects become particularly conspicuous in relation to certain social events. Several sociological and psychological models have been used to examine the theoretical background in studies investigating the impact of a wide range of social events on suicidal behaviour [1-4]. However, these studies have predominantly investigated national events [5-7].

It is well known that the average risk of suicide-related behaviour in certain sub-populations (age-related, professional, gender-related, or religious) may vary substantially [8,9]. Each subpopulation contributes to the general rate of suicide-related behaviour, however, not every sub-population is available for direct evaluation. Some of them are impermanent and changeable (concert audiences or football spectators), and others cannot be easily subjected to investigation (closed professional or religious communities). In such situations, it is necessary to obtain data using other approaches. We hypothesize that, just like national social events that affect the suicide-related behaviour of the entire population, local events confined to sub-populations may also affect the general rate of suicide-related behaviour in local populations. The variation of the latter would theoretically depend on how a mass gathering’s particular milieu affected the risk of suicide-related behaviour in the sub-population of its participants. If so, an analysis of the general population’s suicide-related behaviour rates with respect to mass gatherings might be useful as an indirect evaluation of the level of suicide-related behaviour in a sub-population of people participating in gatherings of various kinds, such as concerts, sports competitions, and religious events.

### Music shows and concerts

To our knowledge, no research has been conducted on the effect of music shows on suicide, and existing studies have only a distant relation to this particular problem. Some lifetime studies have reported that attending certain kinds of cultural events may have a beneficial effect on physical health, social functioning, and vitality [10]. However, one paper describes ‘epidemical mass fainting’ among those attending rock concerts [11].

### Sports events

Sports events appear to be the only kind of mass gatherings whose effects on suicide have been a subject to an epidemiological evaluation [7]. Two earlier studies [12,13] found no significant changes in national suicide rates related to sports events. However, a Canadian study reported mixed results: whereas the general suicide rate was not affected by sports events, young males (15–34 years old) had an increased suicide risk during the hockey playoffs [14]. A later analysis of the effects of professional sports games on suicide in major US cities reported a relationship between the games and suicide rates [15].

As for the effect of a local team’s performance on suicide, a British study reported a significant short-term (12-hour) increase in deliberate self-poisonings after the defeat of a local football team [16]. Another US study found that there were fewer suicides when the US Olympic ice hockey team beat the Soviet Union and on Super Bowl Sundays [17].

All these investigations, however, examined mostly national and international sports events that affected a great part of the population. Moreover, these research findings give no clue as to whether attending a major sports event has an influence on suicide-related behaviour.

### Mass religious events

A number of investigations show that religious festivities (e.g., Christmas) are followed by an increase in the suicide rate [6]. However, practically no research has been conducted on whether mass gatherings carried out by religious organisations influence suicide-related behaviour rates.

At the same time, it is commonly accepted that religiosity is a factor that may decrease the risk of suicide-related behaviour. Durkheim perceived religious affiliation as a proxy for integration and regulation, which in his opinion affected the suicide rate [1]. However, some authors suggest that the correlation between suicide and religious affiliation has disappeared [18]. At the same time, the results of more recent representative cross-national investigations have demonstrated that in countries where religion retains a real social significance, the suicide level is the lowest. However, it must be noted that this phenomenon is typical only in countries that profess traditional religions [19]. Conversely, it is well known that all mass suicides in recent decades were arranged exclusively by members of so-called new religious movements [20].

Although the volume of psychiatric studies on the phenomenon of new religious movements is extremely limited, some studies have, nevertheless, evaluated the level of suicide-related behaviour among former and current devotees of new religious movements.

A study of 68 active devotees of Transcendental Meditation reported that 20% of them expressed serious suicidal tendencies [21]. According to another study, 18.8% of 154 former new religious movement members had suicidal or self-destructive tendencies [22]. Furthermore, a study of 400 former members of new religious movements found that, among other symptoms, 21% of them reported suicidal or self-destructive tendencies [23]. Another study that evaluated 43 former members of the Word of Life new religious movement found that 63% of the group’s members ‘had experienced suicidal thoughts’ and 23% ‘had made a serious suicide attempt’ [24]. It is rather significant that many studies have obtained similar results with regard to the suicide-related behaviour among current and former members of new religious movements.

It follows from the above that certain mass gatherings may influence the suicide rate in the population. The aim of this study is to provide a statistical evaluation of the possible relationship between mass gatherings in some relatively small urban sub-populations and the suicide rates of a major city.

## Methods

### Sample

The data used in this study are from the daily registrations of suicide-related incidents in the industrial city of Dnipropetrovsk, Central Ukraine (approximately 1 million residents) over a period of 6 years, 2005–2010. Data on suicide attempts (Type II, by self-poisoning) were obtained from the Dnipropetrovsk City Centre for Acute Poisoning. The total of 1,078 suicide attempt cases (Х60-Х69 according to the Tenth Revision of the International Classification of Diseases [ICD-10]) included 314 men and 764 women. Data on suicides were obtained from the Dnipropetrovsk Forensic Medical Examination Bureau. There were 1,027 suicides (Х60-Х75, X78, X80, X81, Х83), of which 802 were men and 225 were women. The main methods of suicide were as follows: hanging – 81.8%; jumping off a high place – 9.4%; firearm – 3.6%; sharp object – 3.4%; self-poisoning – 0.7%.

The obtained primary data were arranged as a daily time series. Daily sums of suicide-related behaviour acts for each sex and the total were calculated on the basis of primary data.

All the data used for this study were released with the official approval of the Department of Internal Affairs of the Dnepropetrovsk City Government. The data were impersonalized and non-identifiable.

### Data on mass events

Data on the 427 mass events in Dnipropetrovsk for the years 2005–2010 are shown in Table 1. These events included 88 large concerts and 217 home and guest games of the local football team, Dnipro (playing in the national Premier League; see http://www.fcdnipro.ua/). In addition, our study included 32 games of the national football team in qualifying games for the 2006 World Cup, EURO 2008, and the 2010 World Cup, as well as four games in the 2006 World Cup championship (see http://www.fanatukr.com/). In all cases, we took into account the game results and official data on the number of local citizens who visited the Dnipro games in Dnipropetrovsk, as well as its games elsewhere.

**Table 1:** Mass events that were pertinent to different sub-populations in Dnipropetrovsk city from 2005 to 2010

**Mass events that were pertinent to different sub-populations in Dnipropetrovsk city from 2005 to 2010**		
**Type of mass event**	**Number of events**	**Average participants proportion of urban population (%)**
Matches of local football team:		
At home	105	1.20
Win	53	1.00
Draw	28	1.30
Defeat	24	1.60
Away	112	0.04
Win	45	0.03
Draw	36	0.04
Defeat	31	0.05
Matches of national football team:		
Elimination games	32	n/i
Win	15	n/i
Draw	8	n/i
Defeat	9	n/i
Championship	4	n/i
Win	3	n/i
Defeat	1	n/i
Large concerts	88	0.60
Orthodox Church	8	n/i
New religious movements	78	0.30

*n/i* = no information.

With respect to religion, because the activities of religious organizations typically operate on a weekly basis, we took into account only non-regular, extraordinary gatherings. The Orthodox Church held eight such events to venerate miracle-working relics, which devotees associate with miraculous healing. New religious movements arranged 78 public mass events.

For gatherings where the average number of participants could be determined, it was possible to calculate their proportion with respect to the population of the city. Except for the football matches, no events were broadcast on TV.

### Bivariate analysis

Data on suicide-related behaviour incidents are count variables conforming to the Poisson distribution with the share of zero counts ranging from 40.5 to 90.0%. We attempted to normalize the distribution by substituting the weekly total for the daily data. However, the Kolmogorov-Smirnov test showed that the data are still not normal and therefore non-parametric procedures are appropriate for the analysis.

The statistical evaluation of how a certain event influences the suicide-related behaviour risk poses a methodological problem. Because there is a considerable weekly pattern in the number of suicides [25], one approach that we attempted involves comparing the risks in the 7 days following the event with those in the 7 days preceding the event. For each comparison the *relative risk* (RR) was calculated as the ratio of the number of suicide-related behaviour incidents in a given period to the number of the same incidents in the control period. This is really an odds ratio whose significance can be estimated from a Poisson regression, where the dependent variable was the vector of counts after and before the event. The only independent variable is a two-level factor indicating which group the values came from [26].

Although the comparison of suicide-related behaviour rates before and after events provides a means to establish the association between suicide behaviour and the events, the anticipation of some events may also influence suicide rates [5,6]. Therefore, we also used another approach, which consisted of comparing the average 7-day risks before and after each type of gathering to the average risks during all other days in the temporal sequence. The Mann–Whitney test and the Wilcoxon signed-ranks test for independent samples were used to make the comparisons.

The bivariate analyses and data manipulations were conducted using Microsoft Office Excel 2003 (Microsoft Corp. Redmond, WA, USA), PASW IBM Statistics version 18.0 (IBM, Armonk, NY, USA) and R language (R Foundation for Statistical Computing, Vienna, Austria).

### Multivariate analysis

When bivariate analysis leads to rejection of the null hypothesis, this finding requires an estimation of the potential confounding effects of numerous socio-environmental variables. We, therefore, used the negative binomial regression, which is an appropriate procedure for count data with a large proportion of zero counts.

### Dummy variables of events

The data on all types of mass gatherings and other events were represented as dummy variables: the 7 days of the estimated period were coded as ‘1’ and other days as ‘0’. For each type of regular or pre-announced event, two dummy variables were created: ‘days before the event’ and ‘days after the event’. Football games are represented by four dummy variables: ‘days before the event’, and three more after-the-event variables that coded separately for victories, drawn games, and defeats.

### Covariates

A literature search of the MEDLINE database suggests there is an extensive set of the time-varying factors that may significantly influence suicide-related behaviour. We identified as many of these variables as possible and matched them with our suicide data. An additional file shows these variables and references in more details (see Additional file 1).

However, we did some additional analysis to select those independent variables that might be useful in a multivariate setting. We used Kendall’s tau-b correlation for continuous independent variables and selected only those that had significant correlations with any of the response variables.

With respect to dummy variables, we compared the response variables for the days designated as ‘1’ with those designated as ‘0’ in the procedures of the Mann–Whitney test and the Poisson regression described above. Those variables that yielded a significant result were selected for further model building.

After eliminating some collinear variables, we identified the following independent variables as covariates for model building:

Solar and planetary variables: daily number of sun spots, daily flux of protons >100 Mev, and the average daily Dst geomagnetic index;

Weather-climatic and seasonal variables: minimal daily temperature, change of minimal daily temperature compared with the previous day, daily sunshine time, and ‘month’ as a set of dummy variables (April being the base category);

Socio-economic variables: monthly birth rate in the city, monthly death rate, monthly rate of divorces, monthly inflation index, wage arrears per capita, monthly rate of unemployment, monthly rate of in-migration, monthly rate of international migration, national lottery jackpot in USD, and ‘day of week’ as a set of dummy variables (Friday being the base category);

Mental morbidity: daily urgent hospitalisation for mental treatment (except the cases of suicidal behaviour);

National events: state Orthodox feasts, and general elections.

### Regression modelling

Although we did engage in exploratory analysis of each variable, we produced our models through a data-mining procedure in the R environment [27]. As a first step in that process, we ran a stepwise Poisson regression of a dependent variable against all other independent variables. We ran the step procedure in both directions to account for the multicollinearity of relationships. To ensure the principle of minimal description length, we used Schwartz’s version of the Bayesian information criterion [28]. In the next stage of our analysis, we ran a negative binomial regression to model the overdispersion parameter [29], which turned out to be more adequate than the corresponding standard Poisson models.

Finally, to take into account the fact that we were running regressions on time-series data, we computed robust estimates using the ’sandwich’ approach [30]. The last two stages of the analysis were done in the ‘Zelig’ interface using the ‘negbin’ function [31].

## Results

### Bivariate analysis

Table 2 shows the significant findings obtained when comparing the data on suicide-related behaviour during the 7 days before and after various mass gatherings. Female suicide-related behaviour increased after a football game in the city (RR = 1.17; *p =* 0.045). Male suicide attempts became more likely after a victory in an elimination game (RR = 3.50; *p =* 0.027). Female suicide attempts (RR = 1.29; *p =* 0.012), male suicides (RR = 1.24; *p =* 0.027), and all secondary indices increased after mass events organized by new religious movements.

**Table 2:** Significant relative risks (RR) as ratio of suicidal incidents after and before some mass events

**Significant relative risks (RR) as ratio of suicidal incidents after and before some mass events.**			
	**Football match played in the city (n = 105)**	**Winning an elimination game (n = 15)**	**New religious movement mass event (n = 78)**
	**RR (p)**	**RR (p)**	**RR (p)**
Suicide attempt			
Male	1.14 (0.341)	**3.50(0.027)**	1.06 (0.699)
Female	1.12 (0.192)	0.90 (0.686)	**1.29(0.012)**
Suicides			
Male	0.96 (0.613)	0.87 (0.518)	**1.24(0.027)**
Female	1.36 (0.068)	0.46 (0.117)	0.92 (0.635)
Suicide-related behaviour			
Male	1.01 (0.943)	1.08 (0.695)	**1.19 (0.037)**
Female	**1.17 (0.045)**	0.76 (0.246)	**1.19(0.044)**
Total	1.08 (0.156)	0.93 (0.653)	**1.19(0.004)**

*RR* = relative risk, the odds ratio of suicide-related behaviour incidences for 7 days after the event and 7 days before the event; *p* = probability of the null hypothesis that the odds ratio in the universe is equal to one. Bold type indicates significant findings.

Table 3 shows the significant findings when comparing the suicide-related behaviour risk in the urban population around the period of mass gatherings with that on all other days. We found a significant decrease in female suicide-related behaviour after large concerts (RR = 0.81; *p =* 0.01), as well as a decrease in female suicide-related behaviour and suicide attempt after exhibitions of miracle-working relics (RR = 0.48; *p =* 0.028 and RR = 0.46; *p =* 0.041 respectively).

**Table 3:** Significant relative risk (RR) of suicide-related behaviour around the time of some mass events

**Significant relative risk (RR) of suicidal behaviour around the time of some mass events.**					
	**Post factum concert**	**Post factum home defeat of the local team**	**Before the national team played in a championship**	**Post factum Orthodox mass events**	**Post factum new religious movement mass events**
	**RR (p)**	**RR (p)**	**RR (p)**	**RR (p)**	**RR (p)**
Suicide attempt					
Male	1.07 (0.531)	1.36 (0.158)	2.07 (0.317)	1.00 (0.832)	1.14 (0.185)
Female	0.83 (0.055)	1.03 (0.826)	0.60 (0.281)	**0.46(0.041)**	**1.27(0.004)**
Suicides					
Male	1.08 (0.333)	1.31 (0.093)	0.78 (0.572)	1.00 (0.686)	**1.29(0.006)**
Female	0.73 (0.071)	1.00 (0.807)	**3.60(0.001)**	0.60 (0.381)	1.00 (0.970)
Suicide-Related Behaviour					
Male	1.08 (0.141)	**1.32(0.047)**	1.12 (0.921)	1.00 (0.895)	**1.25(0.019)**
Female	**0.81(0.010)**	1.02 (0.858)	1.27 (0.843)	**0.48(0.028)**	**1.21(0.011)**
Total	0.95 (0.681)	1.18 (0.316)	1.19 (0.486)	0.75 (0.119)	**1.23(0.000)**

*RR* = relative risk, risk of suicidal behaviour in a particular 7-days period compared with other times; *p =* probability according to two-sided Mann–Whitney test and Wilcoxon signed ranks test. Bold type indicates significant findings.

A significant increase was evident for male suicide-related behaviour after the home defeat of the local football team (RR = 1.32; *p =* 0.047) and for female suicides before the national team played a World Cup game (RR = 3.60; *p =* 0.001). After mass events organized by new religious movements, there was a significant increase in female suicide attempts (RR = 1.27; *p =* 0.004), male suicides (RR = 1.29; *p =* 0.006), and secondary indices for both sexes.

### Multivariate analysis

Data contained in Additional file 2 show the results of negative binominal regression modelling. There were no significant covariates in only one case (male suicide attempts). A two types of mass gatherings were included in the models as relevant factors — defeat of the local team at home, and mass events organized by new religious movements. They had significant regression coefficients in the models for three dependent variables — male suicides, male suicide attempts, and total suicide-related behaviour incidents.

In the male suicide model, mass events organized by new religious movements had a significant regression coefficient (beta = 0.255; *p =* 0.002) with the covariates of Sunday (negative) and Monday (positive).

The male suicide-related behaviour model showed significant effects of home defeats and mass events organized by new religious movements. The regression coefficients for defeats and new religious movement events were 0.371 (*p =* 0.002) and 0.251 (*p <* 0.001), respectively. This model also included the covariates of Sunday, Monday, and in-migration.

For the total suicide-related incidents, new religious movement events was a significant factor (beta = 0.187; *p <* 0.001) with the covariates of minimal daily temperature, Sunday, Monday, inflation index, and in-migration.

## Discussion

### Music concerts and shows

A bivariate analysis showed that after concerts, there is a significant decrease in the RR for female suicide-related behaviour of up to 0.81 (Table 3). Although these results correlate with the beneficial effect of cultural events reported by other scholars [10], we had doubts about a straightforward interpretation of our results. It is unlikely that decreasing a risk in a sub-population that amounts to 0.6% of the total population can substantially lower the risk for the latter. Therefore, we are not surprised that our multivariate analysis does not support this result.

### Football matches

In our bivariate analysis, we discovered a significant increase in male suicide attempts in the urban population after victories by the national team in elimination games and in female suicide-related behaviour after home games of the local football team, Dnipro (Table 2). Our multivariate analysis, however, only shows that there is a significant increase in male suicide-related behaviour when the local team was defeated at home (Table 3 and see Additional file 2).

Other studies have reported that suicide-related behaviour rates may rise significantly after the failure of one’s favourite team [14-16]. This has been suggested to result from ‘dysphoric and suicidal moods in individuals who deeply identify themselves with the team’ [15]. Clearly, a fan may view the team as an extension of self [32], and the team’s defeat may be perceived as a personal failure, in some cases reaching clinically significant levels, similar to the impact of traumatic events [33]. All these findings are in agreement with Gabennesch’s theory [2].

However, these findings do not allow for an assessment of the relationship between suicide-related behaviour risk and one’s personal presence at the stadium or watching such events on TV, although it has been suggested that such a relationship may exist [7].

At the same time, the number of fans supporting Dnipro’s guest games was 30 times less than home games (Table 1). As for the games played by the national team, the number of supporters from Dnipropetrovsk was much less. Although all games were broadcast on TV, the two latter types of games were not associated with significant changes in the suicide-related behaviour risks. This may suggest that the increase in the urban suicide-related behaviour rate may be contingent upon fellow fans attending the game.

Unfortunately, ‘there is lack of research focusing on how sports spectatorship might influence levels of suicide risk in individuals, and how mediating variables might operate on the individual level’ [7]. However, the psychological literature on crowds has highlighted that there are two common factors leading to crowd behaviour. First, there must be a ‘seed’, an individual or small group that attempts to engage the crowd. Second, the crowd engages with the seed and has a shared sense of identity [34].

At a football stadium, the seed is fans with a ‘high level of self-identification’ with the team [35]. Therefore, when the crowd engages with the seed, it may temporarily share the high level of self-identification with the team and the defeat of one’s team becomes a significant traumatic event with a ‘broken-promises effect’ for a greater number of people. If such a sub-population is relatively large, it may temporarily increase the suicide-related behaviour rate in the entire population.

It is also possible that an increase in urban suicide-related behaviour rate may be related to engaging in aggressive crowd behaviour and excessive alcohol consumption [35,36], which may lead to escalation of interpersonal conflicts within the family environment [37]. Thus, a recent investigation has asserted that ‘suicide completers were significantly more likely than comparison subjects to have experienced interpersonal conflict in the months leading up to their death’ [38].

It is quite possible that suicide-related behaviour is additionally contingent upon personal harmful events or peculiarities. However, these personal data are not available in our data set. Yet, we find that a significant change of urban male suicide-related behaviour rate takes place when only about 1.6% of the urban population is present at the stadium (Table 1).

### Veneration of miracle-working icons and relics

In our bivariate statistical evaluation, we discovered a significant decrease in the relative risk of female suicide attempts and suicide-related behaviour after extraordinary mass events arranged by the Orthodox Church (Table 3). However, multivariate analysis did not support the results of the bivariate analysis.

### Mass events in new religious movements

A bivariate statistical analysis showed there was a significant increase in almost all suicide-related behaviour rates after extraordinary mass events held by new religious movements (Tables 2 and 3). Multivariate analysis also showed that this factor had a positive effect for the models of male suicides, male suicide-related behaviour, and total suicide-related behaviour. Thus, new religious movement mass events may have a significant relationship to the suicide-related behaviour risk in a population, even though, on average, the participants of such gatherings as a sub-population do not exceed 0.3% of the total population.

Since Durkheim’s time, scholars have believed that religion acts as a powerful counteragent to suicide [1]. More recently, however, it has been hypothesized that there are both ‘life-preserving’ and ‘non-lifesaving’ beliefs. The latter is ‘probably relatively irrelevant to suicide prevention’ [39].

However, our data regarding new religious movement gatherings cannot be explained by the decrease or disappearance of the anti-suicidal effects of religion. Simultaneously, our findings, as well as those of others [21-24], may provide evidence of the existence of some religious cults that may have a negative impact on mental and behavioural aspects of their devotees [40,41]. Moreover, the exclusive relationship of mass suicides with new religious movements [20] indicates that the doctrines and activities of the latter may include some aspects that increase the suicide-related behaviour risk among devotees. They may also allow their leaders to intensify and synchronize such behaviour up to tragic levels.

With regard to possible explanations, we should note that some people may have types of religiosity that correlate positively with various forms of intrapersonal and interpersonal conflict [42]. Moreover, the doctrines of many new religious movements contain provisions aimed at terminating their devotees’ integration with the conventional society, and motivating devotees to cut off relations and instigate conflict with their relatives and friends [40,41]. Undoubtedly, such doctrinal provisions may lead to the utmost individualism, isolation, and intensification of *anomie* with all the consequences described by Durkheim [1] among new religious movement devotees and their relatives.

Furthermore, the doctrines of new religious movements may provoke profound intrapersonal conflicts. Thus, when religious leaders persuade their devotees that divine powers will heal them and produce other instant miracles, it may lead to rapid suicidal effects in devotees owing to an intrapersonal conflict ‘between raised expectancy and stubborn reality’, which are clearly explicable within the framework of Gabennesch's theory of the ‘broken-promise effect’ [2]. At the same time, we do not have enough knowledge about how the specific forms of crowd behaviour in new religious movements may influence the suicide-related behaviour of devotees. For this reason, we may suppose at present that conflict of various kinds may have great explanatory power to suicide-related behaviour.

However, we should not assume that new religious movements make Durkheim’s theory outdated. As we have already noted, this theory is still effective in explaining the suicide level in populations where traditional religions reign supreme [19]. However, the anti-suicidal mechanisms discussed by Durkheim may not apply to groups whose self-identification is based on their contrast to traditional religion and culture.

## Conclusions

We found a significant increase in male and general suicide-related behaviour rates in an urban population after mass gatherings involving sub-populations of football fans and devotees of new religious movements amounting to only 1.6 and 0.3% of the population, respectively. It may suggest a causal link between such events and their participants’ suicide-related behaviour. We are inclined to interpret the findings in terms of the ‘broken-promises effect’ in the context of crowd behaviour. We believe that crowd behaviour in itself may not cause suicidal behaviour, yet it may exacerbate the ‘broken promise effect’ for its participants.

We suspect that the significance of the relationship between such events and population suicide-related behaviour rates may be contingent upon the relative size of the sub-population and its level of suicide-related behaviour. However, only further research may reveal the efficiency of our approach for assessing the level of suicide-related behaviour in a sub-population.

All the findings in this study can be explained largely within the framework of Gabennesch’s suicide theories of the ‘broken-promises effect’. However, we believe that suicide-related behaviour rates in an urban population after some types of mass gathering may also depend on the intensification of intra- and interpersonal conflicts, not only among the event’s participants, but also in their families and social environment.

Our study supports the view that ‘more studies are needed to understand the differing effect of different types of social events on suicidal behaviour among certain subpopulations and to develop suicide prevention approaches effective on both macro (societal) and the individual (psychological) levels’ [7]. Our findings provide new evidence on how some forms of crowd behaviour may influence suicide-related behaviour risk. Otherwise, inadequate evaluation of these processes in some sub-populations may lead to a ‘broken-promise effect’ for the programmes that aim to reduce suicide rates in the general populations.

## Abbreviations

RR: Relative risk; p: Probability; beta: Regression coefficient.

## Competing interests

The authors declare that they have no competing interests.

## Authors’ contributions

VSU conceived of the study and its design, carried out statistical analysis and coordination, and helped draft the manuscript. SNS conceived of the study and interpreted the data. YNS conducted the acquisition and interpretation of the data. EDP conducted the regression analysis and made critical revisions. All the authors read and approved the final manuscript.

## Authors’ information

VSU: MD, PhD.

SNS: MD, Senior Psychiatrist of emergency medical aid.

YNS: Member of Dnipropetrovsk Municipal Expert Advisory Committee for Protection of Citizens’ Constitutional Rights.

EDP: PhD (Sociology, University of Michigan, 1996), director of the Laboratory for Comparative Social Research at the Higher School of Economics, Petersburg, Russia (http://lcsr.hse.ru/en/), member of the Executive Board of the World Values Survey Association, the World Values Survey representative in Russia.

## Pre-publication history

The pre-publication history for this paper can be accessed here:

http://www.biomedcentral.com/1471-2458/14/308/prepub

## Supplementary Material

Additional file 1 References for independent variables considered as covariates for model building. Click here for file

Additional file 2: Negative binominal regression models for the rates of suicidal behaviour in an urban population. Click here for file
