# Supplementary material for: Impact of some types of mass gatherings on current suicide risk in an urban population: statistical and negative binominal regression analysis of time series
Source: BMC Public Health. 2014 Apr 4;14:308. doi: 10.1186/1471-2458-14-308 (PMC4021406; doi:10.1186/1471-2458-14-308)
Supplement: Additional file 1 — References for independent variables considered as covariates for model building. [file 1471-2458-14-308-S1.doc]

**Additional file 1:** **References for independent variables considered as covariates for model building.**

| **References for independent variables considered as covariates for model building** | |
| --- | --- |
| **Covariate** | **Reference** |
| **Solar and planetary** | |
| **daily number of sun spots** | [Otsu A](http://www.ncbi.nlm.nih.gov/sites/entrez?Db=pubmed&Cmd=Search&Term="Otsu A"%5BAuthor%5D&itool=EntrezSystem2.PEntrez.Pubmed.Pubmed_ResultsPanel.Pubmed_DiscoveryPanel.Pubmed_RVAbstractPlus), [Chinami M](http://www.ncbi.nlm.nih.gov/sites/entrez?Db=pubmed&Cmd=Search&Term="Chinami M"%5BAuthor%5D&itool=EntrezSystem2.PEntrez.Pubmed.Pubmed_ResultsPanel.Pubmed_DiscoveryPanel.Pubmed_RVAbstractPlus), [Morgenthale S](http://www.ncbi.nlm.nih.gov/sites/entrez?Db=pubmed&Cmd=Search&Term="Morgenthale S"%5BAuthor%5D&itool=EntrezSystem2.PEntrez.Pubmed.Pubmed_ResultsPanel.Pubmed_DiscoveryPanel.Pubmed_RVAbstractPlus), [Kaneko Y](http://www.ncbi.nlm.nih.gov/sites/entrez?Db=pubmed&Cmd=Search&Term="Kaneko Y"%5BAuthor%5D&itool=EntrezSystem2.PEntrez.Pubmed.Pubmed_ResultsPanel.Pubmed_DiscoveryPanel.Pubmed_RVAbstractPlus), [Fujita D](http://www.ncbi.nlm.nih.gov/sites/entrez?Db=pubmed&Cmd=Search&Term="Fujita D"%5BAuthor%5D&itool=EntrezSystem2.PEntrez.Pubmed.Pubmed_ResultsPanel.Pubmed_DiscoveryPanel.Pubmed_RVAbstractPlus), [Shirakawa T](http://www.ncbi.nlm.nih.gov/sites/entrez?Db=pubmed&Cmd=Search&Term="Shirakawa T"%5BAuthor%5D&itool=EntrezSystem2.PEntrez.Pubmed.Pubmed_ResultsPanel.Pubmed_DiscoveryPanel.Pubmed_RVAbstractPlus): **Correlations for number of sunspots, unemployment rate, and suicide mortality in Japan.** [*Percept Mot Skills.*](javascript:AL_get(this, 'jour', 'Percept Mot Skills.');) 2006, **102**:603-8.  [Stoupel E](http://www.ncbi.nlm.nih.gov/sites/entrez?Db=pubmed&Cmd=Search&Term="Stoupel E"%5BAuthor%5D&itool=EntrezSystem2.PEntrez.Pubmed.Pubmed_ResultsPanel.Pubmed_DiscoveryPanel.Pubmed_RVAbstractPlus), [Kalëdienë R](http://www.ncbi.nlm.nih.gov/sites/entrez?Db=pubmed&Cmd=Search&Term="Kalëdienë R"%5BAuthor%5D&itool=EntrezSystem2.PEntrez.Pubmed.Pubmed_ResultsPanel.Pubmed_DiscoveryPanel.Pubmed_RVAbstractPlus), [Petrauskiene J](http://www.ncbi.nlm.nih.gov/sites/entrez?Db=pubmed&Cmd=Search&Term="Petrauskiene J"%5BAuthor%5D&itool=EntrezSystem2.PEntrez.Pubmed.Pubmed_ResultsPanel.Pubmed_DiscoveryPanel.Pubmed_RVAbstractPlus), [Starkuviene S](http://www.ncbi.nlm.nih.gov/sites/entrez?Db=pubmed&Cmd=Search&Term="Starkuviene S"%5BAuthor%5D&itool=EntrezSystem2.PEntrez.Pubmed.Pubmed_ResultsPanel.Pubmed_DiscoveryPanel.Pubmed_RVAbstractPlus), [Abramson E](http://www.ncbi.nlm.nih.gov/sites/entrez?Db=pubmed&Cmd=Search&Term="Abramson E"%5BAuthor%5D&itool=EntrezSystem2.PEntrez.Pubmed.Pubmed_ResultsPanel.Pubmed_DiscoveryPanel.Pubmed_RVAbstractPlus), [Israelevich P](http://www.ncbi.nlm.nih.gov/sites/entrez?Db=pubmed&Cmd=Search&Term="Israelevich P"%5BAuthor%5D&itool=EntrezSystem2.PEntrez.Pubmed.Pubmed_ResultsPanel.Pubmed_DiscoveryPanel.Pubmed_RVAbstractPlus), [Sulkes J](http://www.ncbi.nlm.nih.gov/sites/entrez?Db=pubmed&Cmd=Search&Term="Sulkes J"%5BAuthor%5D&itool=EntrezSystem2.PEntrez.Pubmed.Pubmed_ResultsPanel.Pubmed_DiscoveryPanel.Pubmed_RVAbstractPlus): **Suicide-homicide temporal interrelationship, links with other fatalities, and environmental physical activity.** [*Crisis*](javascript:AL_get(this, 'jour', 'Crisis.');) 2005, **26**:85-89. |
| **daily flux of protons >100 Mev** | [Stoupel E](http://www.ncbi.nlm.nih.gov/sites/entrez?Db=pubmed&Cmd=Search&Term="Stoupel E"%5BAuthor%5D&itool=EntrezSystem2.PEntrez.Pubmed.Pubmed_ResultsPanel.Pubmed_DiscoveryPanel.Pubmed_RVAbstractPlus), [Israelevich P](http://www.ncbi.nlm.nih.gov/sites/entrez?Db=pubmed&Cmd=Search&Term="Israelevich P"%5BAuthor%5D&itool=EntrezSystem2.PEntrez.Pubmed.Pubmed_ResultsPanel.Pubmed_DiscoveryPanel.Pubmed_RVAbstractPlus), [Gabbay U](http://www.ncbi.nlm.nih.gov/sites/entrez?Db=pubmed&Cmd=Search&Term="Gabbay U"%5BAuthor%5D&itool=EntrezSystem2.PEntrez.Pubmed.Pubmed_ResultsPanel.Pubmed_DiscoveryPanel.Pubmed_RVAbstractPlus), [Abramson E](http://www.ncbi.nlm.nih.gov/sites/entrez?Db=pubmed&Cmd=Search&Term="Abramson E"%5BAuthor%5D&itool=EntrezSystem2.PEntrez.Pubmed.Pubmed_ResultsPanel.Pubmed_DiscoveryPanel.Pubmed_RVAbstractPlus), [Petrauskiene J](http://www.ncbi.nlm.nih.gov/sites/entrez?Db=pubmed&Cmd=Search&Term="Petrauskiene J"%5BAuthor%5D&itool=EntrezSystem2.PEntrez.Pubmed.Pubmed_ResultsPanel.Pubmed_DiscoveryPanel.Pubmed_RVAbstractPlus), [Kalediene B](http://www.ncbi.nlm.nih.gov/sites/entrez?Db=pubmed&Cmd=Search&Term="Kalediene B"%5BAuthor%5D&itool=EntrezSystem2.PEntrez.Pubmed.Pubmed_ResultsPanel.Pubmed_DiscoveryPanel.Pubmed_RVAbstractPlus), [Domarkiene S](http://www.ncbi.nlm.nih.gov/sites/entrez?Db=pubmed&Cmd=Search&Term="Domarkiene S"%5BAuthor%5D&itool=EntrezSystem2.PEntrez.Pubmed.Pubmed_ResultsPanel.Pubmed_DiscoveryPanel.Pubmed_RVAbstractPlus), [Sulkes J](http://www.ncbi.nlm.nih.gov/sites/entrez?Db=pubmed&Cmd=Search&Term="Sulkes J"%5BAuthor%5D&itool=EntrezSystem2.PEntrez.Pubmed.Pubmed_ResultsPanel.Pubmed_DiscoveryPanel.Pubmed_RVAbstractPlus): **Correlation of two levels of space proton flux with monthly distribution of deaths from cardiovascular disease and suicide.** *J Basic Clin Physiol Pharmacol* 2000, **11**:63-71. |
| **Solar radio flux 10.7cm** | [Ruuhela R](http://www.ncbi.nlm.nih.gov/pubmed?term=Ruuhela R%5BAuthor%5D&cauthor=true&cauthor_uid=19101735), [Hiltunen L](http://www.ncbi.nlm.nih.gov/pubmed?term=Hiltunen L%5BAuthor%5D&cauthor=true&cauthor_uid=19101735), [Venäläinen A](http://www.ncbi.nlm.nih.gov/pubmed?term=Venäläinen A%5BAuthor%5D&cauthor=true&cauthor_uid=19101735), [Pirinen P](http://www.ncbi.nlm.nih.gov/pubmed?term=Pirinen P%5BAuthor%5D&cauthor=true&cauthor_uid=19101735), [Partonen T](http://www.ncbi.nlm.nih.gov/pubmed?term=Partonen T%5BAuthor%5D&cauthor=true&cauthor_uid=19101735): **Climate impact on suicide rates in Finland from 1971 to 2003.** *[Int J Biometeorol](http://www.ncbi.nlm.nih.gov/pubmed/19101735#%23)* 2009, **53**:167-75.  [Stoupel E](http://www.ncbi.nlm.nih.gov/sites/entrez?Db=pubmed&Cmd=Search&Term="Stoupel E"%5BAuthor%5D&itool=EntrezSystem2.PEntrez.Pubmed.Pubmed_ResultsPanel.Pubmed_DiscoveryPanel.Pubmed_RVAbstractPlus), [Kalëdienë R](http://www.ncbi.nlm.nih.gov/sites/entrez?Db=pubmed&Cmd=Search&Term="Kalëdienë R"%5BAuthor%5D&itool=EntrezSystem2.PEntrez.Pubmed.Pubmed_ResultsPanel.Pubmed_DiscoveryPanel.Pubmed_RVAbstractPlus), [Petrauskiene J](http://www.ncbi.nlm.nih.gov/sites/entrez?Db=pubmed&Cmd=Search&Term="Petrauskiene J"%5BAuthor%5D&itool=EntrezSystem2.PEntrez.Pubmed.Pubmed_ResultsPanel.Pubmed_DiscoveryPanel.Pubmed_RVAbstractPlus), [Starkuviene S](http://www.ncbi.nlm.nih.gov/sites/entrez?Db=pubmed&Cmd=Search&Term="Starkuviene S"%5BAuthor%5D&itool=EntrezSystem2.PEntrez.Pubmed.Pubmed_ResultsPanel.Pubmed_DiscoveryPanel.Pubmed_RVAbstractPlus), [Abramson E](http://www.ncbi.nlm.nih.gov/sites/entrez?Db=pubmed&Cmd=Search&Term="Abramson E"%5BAuthor%5D&itool=EntrezSystem2.PEntrez.Pubmed.Pubmed_ResultsPanel.Pubmed_DiscoveryPanel.Pubmed_RVAbstractPlus), [Israelevich P](http://www.ncbi.nlm.nih.gov/sites/entrez?Db=pubmed&Cmd=Search&Term="Israelevich P"%5BAuthor%5D&itool=EntrezSystem2.PEntrez.Pubmed.Pubmed_ResultsPanel.Pubmed_DiscoveryPanel.Pubmed_RVAbstractPlus), [Sulkes J](http://www.ncbi.nlm.nih.gov/sites/entrez?Db=pubmed&Cmd=Search&Term="Sulkes J"%5BAuthor%5D&itool=EntrezSystem2.PEntrez.Pubmed.Pubmed_ResultsPanel.Pubmed_DiscoveryPanel.Pubmed_RVAbstractPlus): **Suicide-homicide temporal interrelationship, links with other fatalities, and environmental physical activity.** [*Crisis*](javascript:AL_get(this, 'jour', 'Crisis.');) 2005, **26**:85-89. |
| **the average daily Dst geomagnetic index** | [Stoupel E](http://www.ncbi.nlm.nih.gov/sites/entrez?Db=pubmed&Cmd=Search&Term="Stoupel E"%5BAuthor%5D&itool=EntrezSystem2.PEntrez.Pubmed.Pubmed_ResultsPanel.Pubmed_DiscoveryPanel.Pubmed_RVAbstractPlus), [Kalediene R](http://www.ncbi.nlm.nih.gov/sites/entrez?Db=pubmed&Cmd=Search&Term="Kalediene R"%5BAuthor%5D&itool=EntrezSystem2.PEntrez.Pubmed.Pubmed_ResultsPanel.Pubmed_DiscoveryPanel.Pubmed_RVAbstractPlus), [Petrauskiene J](http://www.ncbi.nlm.nih.gov/sites/entrez?Db=pubmed&Cmd=Search&Term="Petrauskiene J"%5BAuthor%5D&itool=EntrezSystem2.PEntrez.Pubmed.Pubmed_ResultsPanel.Pubmed_DiscoveryPanel.Pubmed_RVAbstractPlus), [Domarkiene S](http://www.ncbi.nlm.nih.gov/sites/entrez?Db=pubmed&Cmd=Search&Term="Domarkiene S"%5BAuthor%5D&itool=EntrezSystem2.PEntrez.Pubmed.Pubmed_ResultsPanel.Pubmed_DiscoveryPanel.Pubmed_RVAbstractPlus), [Radishauskas R](http://www.ncbi.nlm.nih.gov/sites/entrez?Db=pubmed&Cmd=Search&Term="Radishauskas R"%5BAuthor%5D&itool=EntrezSystem2.PEntrez.Pubmed.Pubmed_ResultsPanel.Pubmed_DiscoveryPanel.Pubmed_RVAbstractPlus), [Abramson E](http://www.ncbi.nlm.nih.gov/sites/entrez?Db=pubmed&Cmd=Search&Term="Abramson E"%5BAuthor%5D&itool=EntrezSystem2.PEntrez.Pubmed.Pubmed_ResultsPanel.Pubmed_DiscoveryPanel.Pubmed_RVAbstractPlus), [Israelevich P](http://www.ncbi.nlm.nih.gov/sites/entrez?Db=pubmed&Cmd=Search&Term="Israelevich P"%5BAuthor%5D&itool=EntrezSystem2.PEntrez.Pubmed.Pubmed_ResultsPanel.Pubmed_DiscoveryPanel.Pubmed_RVAbstractPlus), [Sulkes J](http://www.ncbi.nlm.nih.gov/sites/entrez?Db=pubmed&Cmd=Search&Term="Sulkes J"%5BAuthor%5D&itool=EntrezSystem2.PEntrez.Pubmed.Pubmed_ResultsPanel.Pubmed_DiscoveryPanel.Pubmed_RVAbstractPlus): **Three kinds of cosmophysical activity: links to temporal distribution of deaths and occurrence of acute myocardial infarction.** *Med Sci Monit* 2004, **10**:CR80-84. |
| **Weather-climatic and seasonal** | |
| **minimal daily temperature** | Barker A, Hawton K, Fagg J, Jennison C**: Seasonal and weather factors in parasuicide.** *Brit J Psychiatry* 1994, **165**:375-380 |
| **change of minimal daily temperature compared with the previous day** | [Volpe FM](http://www.ncbi.nlm.nih.gov/sites/entrez?Db=pubmed&Cmd=Search&Term="Volpe FM"%5BAuthor%5D&itool=EntrezSystem2.PEntrez.Pubmed.Pubmed_ResultsPanel.Pubmed_DiscoveryPanel.Pubmed_RVAbstractPlus), [Tavares A](http://www.ncbi.nlm.nih.gov/sites/entrez?Db=pubmed&Cmd=Search&Term="Tavares A"%5BAuthor%5D&itool=EntrezSystem2.PEntrez.Pubmed.Pubmed_ResultsPanel.Pubmed_DiscoveryPanel.Pubmed_RVAbstractPlus), [Del Porto JA](http://www.ncbi.nlm.nih.gov/sites/entrez?Db=pubmed&Cmd=Search&Term="Del Porto JA"%5BAuthor%5D&itool=EntrezSystem2.PEntrez.Pubmed.Pubmed_ResultsPanel.Pubmed_DiscoveryPanel.Pubmed_RVAbstractPlus): **Seasonality of three dimensions of mania: psychosis, aggression and suicidality.** [*J Affect Disord*](javascript:AL_get(this, 'jour', 'J Affect Disord.');) 2008, **108**:95-100. |
| **average daily relative humidity (%)** | [Inoue K](http://www.ncbi.nlm.nih.gov/pubmed?term=Inoue K%5BAuthor%5D&cauthor=true&cauthor_uid=23441378), [Nishimura Y](http://www.ncbi.nlm.nih.gov/pubmed?term=Nishimura Y%5BAuthor%5D&cauthor=true&cauthor_uid=23441378), [Fujita Y](http://www.ncbi.nlm.nih.gov/pubmed?term=Fujita Y%5BAuthor%5D&cauthor=true&cauthor_uid=23441378), [Ono Y](http://www.ncbi.nlm.nih.gov/pubmed?term=Ono Y%5BAuthor%5D&cauthor=true&cauthor_uid=23441378), [Fukunaga T](http://www.ncbi.nlm.nih.gov/pubmed?term=Fukunaga T%5BAuthor%5D&cauthor=true&cauthor_uid=23441378): **The relationship between suicide and five climate issues in a large-scale and long-term study in Japan.** *[West Indian Med J](http://www.ncbi.nlm.nih.gov/pubmed/23441378#%23)* 2012, **61**:532-537. |
| **average daily atmospheric pressure (Gpa)** | [Hiltunen L](http://www.ncbi.nlm.nih.gov/pubmed?term=Hiltunen L%5BAuthor%5D&cauthor=true&cauthor_uid=22278192), [Ruuhela R](http://www.ncbi.nlm.nih.gov/pubmed?term=Ruuhela R%5BAuthor%5D&cauthor=true&cauthor_uid=22278192), [Ostamo A](http://www.ncbi.nlm.nih.gov/pubmed?term=Ostamo A%5BAuthor%5D&cauthor=true&cauthor_uid=22278192), [Lönnqvist J](http://www.ncbi.nlm.nih.gov/pubmed?term=Lönnqvist J%5BAuthor%5D&cauthor=true&cauthor_uid=22278192), [Suominen K](http://www.ncbi.nlm.nih.gov/pubmed?term=Suominen K%5BAuthor%5D&cauthor=true&cauthor_uid=22278192), [Partonen T](http://www.ncbi.nlm.nih.gov/pubmed?term=Partonen T%5BAuthor%5D&cauthor=true&cauthor_uid=22278192): Atmospheric **pressure and suicide attempts in Helsinki, Finland.** *[Int J Biometeorol](http://www.ncbi.nlm.nih.gov/pubmed/22278192#%23)* 2012, **56**:1045-1053. |
| **daily atmospheric precipitation (mm)** | [Ruuhela R](http://www.ncbi.nlm.nih.gov/pubmed?term=Ruuhela R%5BAuthor%5D&cauthor=true&cauthor_uid=19101735), [Hiltunen L](http://www.ncbi.nlm.nih.gov/pubmed?term=Hiltunen L%5BAuthor%5D&cauthor=true&cauthor_uid=19101735), [Venäläinen A](http://www.ncbi.nlm.nih.gov/pubmed?term=Venäläinen A%5BAuthor%5D&cauthor=true&cauthor_uid=19101735), [Pirinen P](http://www.ncbi.nlm.nih.gov/pubmed?term=Pirinen P%5BAuthor%5D&cauthor=true&cauthor_uid=19101735), [Partonen T](http://www.ncbi.nlm.nih.gov/pubmed?term=Partonen T%5BAuthor%5D&cauthor=true&cauthor_uid=19101735): **Climate impact on suicide rates in Finland from 1971 to 2003.** *[Int J Biometeorol](http://www.ncbi.nlm.nih.gov/pubmed/19101735#%23)* 2009, **53**:167-175. |
| **daylight hours** | [Hiltunen L](http://www.ncbi.nlm.nih.gov/pubmed?term=Hiltunen L%5BAuthor%5D&cauthor=true&cauthor_uid=21943377), [Suominen K](http://www.ncbi.nlm.nih.gov/pubmed?term=Suominen K%5BAuthor%5D&cauthor=true&cauthor_uid=21943377), [Lönnqvist J](http://www.ncbi.nlm.nih.gov/pubmed?term=Lönnqvist J%5BAuthor%5D&cauthor=true&cauthor_uid=21943377), [Partonen T](http://www.ncbi.nlm.nih.gov/pubmed?term=Partonen T%5BAuthor%5D&cauthor=true&cauthor_uid=21943377): **Relationship between daylength and suicide in Finland.** *[J Circadian Rhythms](http://www.ncbi.nlm.nih.gov/pubmed/21943377#%23)* 2011, 23:10. |
| **daily sunshine time** | [Nejar KA](http://www.ncbi.nlm.nih.gov/sites/entrez?Db=pubmed&Cmd=Search&Term="Nejar KA"%5BAuthor%5D&itool=EntrezSystem2.PEntrez.Pubmed.Pubmed_ResultsPanel.Pubmed_DiscoveryPanel.Pubmed_RVAbstractPlus), [Benseñor IM](http://www.ncbi.nlm.nih.gov/sites/entrez?Db=pubmed&Cmd=Search&Term="Benseñor IM"%5BAuthor%5D&itool=EntrezSystem2.PEntrez.Pubmed.Pubmed_ResultsPanel.Pubmed_DiscoveryPanel.Pubmed_RVAbstractPlus), [Lotufo PA](http://www.ncbi.nlm.nih.gov/sites/entrez?Db=pubmed&Cmd=Search&Term="Lotufo PA"%5BAuthor%5D&itool=EntrezSystem2.PEntrez.Pubmed.Pubmed_ResultsPanel.Pubmed_DiscoveryPanel.Pubmed_RVAbstractPlus): **Sunshine and suicide at the tropic of Capricorn, São Paulo, Brazil, 1996-2004.** [*Rev Saude Publica*](javascript:AL_get(this, 'jour', 'Rev Saude Publica.');) 2007, **41**:1062-1064.  [Maes M](http://www.ncbi.nlm.nih.gov/pubmed?term=Maes M%5BAuthor%5D&cauthor=true&cauthor_uid=7872046), [De Meyer F](http://www.ncbi.nlm.nih.gov/pubmed?term=De Meyer F%5BAuthor%5D&cauthor=true&cauthor_uid=7872046), [Thompson P](http://www.ncbi.nlm.nih.gov/pubmed?term=Thompson P%5BAuthor%5D&cauthor=true&cauthor_uid=7872046), [Peeters D](http://www.ncbi.nlm.nih.gov/pubmed?term=Peeters D%5BAuthor%5D&cauthor=true&cauthor_uid=7872046), [Cosyns P](http://www.ncbi.nlm.nih.gov/pubmed?term=Cosyns P%5BAuthor%5D&cauthor=true&cauthor_uid=7872046). **Synchronized annual rhythms in violent suicide rate, ambient temperature and the light-dark span.** *[Acta Psychiatr Scand](http://www.ncbi.nlm.nih.gov/pubmed/7872046#%23)* 1994, **90**:391-396. |
| **set of dummy variables ‘month’ (reference category, April)** | [Partonen T](http://www.ncbi.nlm.nih.gov/sites/entrez?Db=pubmed&Cmd=Search&Term="Partonen T"%5BAuthor%5D&itool=EntrezSystem2.PEntrez.Pubmed.Pubmed_ResultsPanel.Pubmed_DiscoveryPanel.Pubmed_RVAbstractPlus), [Haukka J](http://www.ncbi.nlm.nih.gov/sites/entrez?Db=pubmed&Cmd=Search&Term="Haukka J"%5BAuthor%5D&itool=EntrezSystem2.PEntrez.Pubmed.Pubmed_ResultsPanel.Pubmed_DiscoveryPanel.Pubmed_RVAbstractPlus), [Nevanlinna H](http://www.ncbi.nlm.nih.gov/sites/entrez?Db=pubmed&Cmd=Search&Term="Nevanlinna H"%5BAuthor%5D&itool=EntrezSystem2.PEntrez.Pubmed.Pubmed_ResultsPanel.Pubmed_DiscoveryPanel.Pubmed_RVAbstractPlus), [Lönnqvist J](http://www.ncbi.nlm.nih.gov/sites/entrez?Db=pubmed&Cmd=Search&Term="Lönnqvist J"%5BAuthor%5D&itool=EntrezSystem2.PEntrez.Pubmed.Pubmed_ResultsPanel.Pubmed_DiscoveryPanel.Pubmed_RVAbstractPlus). **Analysis of the seasonal pattern in suicide.** [*J Affect Disord*](javascript:AL_get(this, 'jour', 'J Affect Disord.');) 2004, **81**:133-139. |
| **Socio-economic** | |
| **monthly birth rate in the city** | Hong J, Knapp M: [**Geographical inequalities in suicide rates and area deprivation in South Korea.**](http://www.ncbi.nlm.nih.gov/pubmed/24327481) *J Ment Health Policy Econ* 2013, **16**:109-119.  Papadopoulos FC, Karamanis G, Brandt L, Ekbom A, Ekselius L: [**Childbearing and mortality among women with anorexia nervosa.**](http://www.ncbi.nlm.nih.gov/pubmed/22887053) *Int J Eat Disord* 2013, **46**:164-170. |
| **monthly death rate** | Harrison DP, Stritzke WG, Fay N, Ellison TM, Hudaib AR: [**Probing the Implicit Suicidal Mind: Does the Death/Suicide Implicit Association Test Reveal a Desire to Die, or a Diminished Desire to Live?**](http://www.ncbi.nlm.nih.gov/pubmed/24611787) *Psychol Assess* 2014, Mar 10. [Epub ahead of print]  Liu RT, Miller I: [**Life events and suicidal ideation and behavior: A systematic review.**](http://www.ncbi.nlm.nih.gov/pubmed/24534642) *Clin Psychol Rev* 2014, **34**:181-192. |
| **monthly infant mortality rate** | Lichtenthal WG, Neimeyer RA, Currier JM, Roberts K, Jordan N: [**Cause of death and the quest for meaning after the loss of a child.**](http://www.ncbi.nlm.nih.gov/pubmed/24520890) *Death Stud* 2013, **37**:311-42. |
| **monthly rate of divorces** | Hong J, Knapp M: [**Geographical inequalities in suicide rates and area deprivation in South Korea.**](http://www.ncbi.nlm.nih.gov/pubmed/24327481) *J Ment Health Policy Econ* 2013, **16**:109-19. |
| **monthly inflation index** | Solano P, Pizzorno E, Gallina AM, Mattei C, Gabrielli F, Kayman J: [**Employment status, inflation and suicidal behaviour: an analysis of a stratified sample in Italy.**](http://www.ncbi.nlm.nih.gov/pubmed/21813481) *Int J Soc Psychiatry* 2012, **58**:477-484. |
| **average monthly wage in hryvnia** | Bezerra Filho JG, Werneck GL, Almeida RL, Oliveira MI, Magalhães FB: [**[Socio-demographic determinants of suicide in the State of Rio de Janeiro, Brazil, 1998-2002].**](http://www.ncbi.nlm.nih.gov/pubmed/22641507) *Cad Saude Publica* 2012, **28**:833-844. |
| **wage arrears per capita** | [Perlman F](http://www.ncbi.nlm.nih.gov/pubmed?term=Perlman F%5BAuthor%5D&cauthor=true&cauthor_uid=19696378)1, [Bobak M](http://www.ncbi.nlm.nih.gov/pubmed?term=Bobak M%5BAuthor%5D&cauthor=true&cauthor_uid=19696378): **Assessing the contribution of unstable employment to mortality in posttransition Russia: prospective individual-level analyses from the Russian longitudinal monitoring survey.** *[Am J Public Health](http://www.ncbi.nlm.nih.gov/pubmed/?term=wage+arrears#%23)* 2009, **99**:1818-1825. |
| **monthly rate of unemployment** | [Otsu A](http://www.ncbi.nlm.nih.gov/sites/entrez?Db=pubmed&Cmd=Search&Term="Otsu A"%5BAuthor%5D&itool=EntrezSystem2.PEntrez.Pubmed.Pubmed_ResultsPanel.Pubmed_DiscoveryPanel.Pubmed_RVAbstractPlus), [Chinami M](http://www.ncbi.nlm.nih.gov/sites/entrez?Db=pubmed&Cmd=Search&Term="Chinami M"%5BAuthor%5D&itool=EntrezSystem2.PEntrez.Pubmed.Pubmed_ResultsPanel.Pubmed_DiscoveryPanel.Pubmed_RVAbstractPlus), [Morgenthale S](http://www.ncbi.nlm.nih.gov/sites/entrez?Db=pubmed&Cmd=Search&Term="Morgenthale S"%5BAuthor%5D&itool=EntrezSystem2.PEntrez.Pubmed.Pubmed_ResultsPanel.Pubmed_DiscoveryPanel.Pubmed_RVAbstractPlus), [Kaneko Y](http://www.ncbi.nlm.nih.gov/sites/entrez?Db=pubmed&Cmd=Search&Term="Kaneko Y"%5BAuthor%5D&itool=EntrezSystem2.PEntrez.Pubmed.Pubmed_ResultsPanel.Pubmed_DiscoveryPanel.Pubmed_RVAbstractPlus), [Fujita D](http://www.ncbi.nlm.nih.gov/sites/entrez?Db=pubmed&Cmd=Search&Term="Fujita D"%5BAuthor%5D&itool=EntrezSystem2.PEntrez.Pubmed.Pubmed_ResultsPanel.Pubmed_DiscoveryPanel.Pubmed_RVAbstractPlus), [Shirakawa T](http://www.ncbi.nlm.nih.gov/sites/entrez?Db=pubmed&Cmd=Search&Term="Shirakawa T"%5BAuthor%5D&itool=EntrezSystem2.PEntrez.Pubmed.Pubmed_ResultsPanel.Pubmed_DiscoveryPanel.Pubmed_RVAbstractPlus): **Correlations for number of sunspots, unemployment rate, and suicide mortality in Japan.** [*Percept Mot Skills*](javascript:AL_get(this, 'jour', 'Percept Mot Skills.');) 2006, **102**:603-608  Solano P, Pizzorno E, Gallina AM, Mattei C, Gabrielli F, Kayman J: [**Employment status, inflation and suicidal behaviour: an analysis of a stratified sample in Italy.**](http://www.ncbi.nlm.nih.gov/pubmed/21813481) *Int J Soc Psychiatry* 2012, **58**:477-484. |
| **monthly rate of marriage** | [Korosec Jagodic H](http://www.ncbi.nlm.nih.gov/pubmed?term=Korosec Jagodic H%5BAuthor%5D&cauthor=true&cauthor_uid=24170723), [Rokavec T](http://www.ncbi.nlm.nih.gov/pubmed?term=Rokavec T%5BAuthor%5D&cauthor=true&cauthor_uid=24170723), [Agius M](http://www.ncbi.nlm.nih.gov/pubmed?term=Agius M%5BAuthor%5D&cauthor=true&cauthor_uid=24170723), [Pregelj P](http://www.ncbi.nlm.nih.gov/pubmed?term=Pregelj P%5BAuthor%5D&cauthor=true&cauthor_uid=24170723): **Availability of mental health service providers and suicide rates in Slovenia: a nationwide ecological study.** *[Croat Med J](http://www.ncbi.nlm.nih.gov/pubmed/24170723#%23)* 2013, **54**:444-452. |
| **monthly rate of in-migration** | Termorshuizen F, Wierdsma AI, Visser E, Drukker M, Sytema S, Laan W, Smeets HM, Selten JP: [**Psychosis and suicide risk by ethnic origin and history of migration in the Netherlands.**](http://www.ncbi.nlm.nih.gov/pubmed/22521494) *Schizophr Res* 2012, 138:268-273. |
| **monthly rate of international migration** | Iliceto P, Pompili M, Candilera G, Borges G, Lamis DA, Serafini G, Girardi P: [**Suicide risk and psychopathology in immigrants: a multi-group confirmatory factor analysis.**](http://www.ncbi.nlm.nih.gov/pubmed/23096150) *Soc Psychiatry Psychiatr Epidemiol* 2013, **48**:1105-1114. |
| **change in dollar rate in cash compared with the previous day** | Solano P, Pizzorno E, Gallina AM, Mattei C, Gabrielli F, Kayman J: [**Employment status, inflation and suicidal behaviour: an analysis of a stratified sample in Italy.**](http://www.ncbi.nlm.nih.gov/pubmed/21813481) *Int J Soc Psychiatry* 2012, **58**:477-484. |
| **national lottery jackpot in USD** | Chen VC, Stewart R, Lee CT: [**Weekly lottery sales volume and suicide numbers: a time series analysis on national data from Taiwan.**](http://www.ncbi.nlm.nih.gov/pubmed/21681453) *Soc Psychiatry Psychiatr Epidemiol* 2012, **47**:1055-1059. |
| **set of dummy variables ‘day of week’ (reference category, Friday)** | [Bozsonyi K](http://www.ncbi.nlm.nih.gov/sites/entrez?Db=pubmed&Cmd=Search&Term="Bozsonyi K"%5BAuthor%5D&itool=EntrezSystem2.PEntrez.Pubmed.Pubmed_ResultsPanel.Pubmed_DiscoveryPanel.Pubmed_RVAbstractPlus), [Veres E](http://www.ncbi.nlm.nih.gov/sites/entrez?Db=pubmed&Cmd=Search&Term="Veres E"%5BAuthor%5D&itool=EntrezSystem2.PEntrez.Pubmed.Pubmed_ResultsPanel.Pubmed_DiscoveryPanel.Pubmed_RVAbstractPlus), [Zonda T](http://www.ncbi.nlm.nih.gov/sites/entrez?Db=pubmed&Cmd=Search&Term="Zonda T"%5BAuthor%5D&itool=EntrezSystem2.PEntrez.Pubmed.Pubmed_ResultsPanel.Pubmed_DiscoveryPanel.Pubmed_RVAbstractPlus): [**The effect of public holidays on the suicide drive (frequency) in Hungary (1970-2002)**]. [*Psychiatr Hung*](javascript:AL_get(this, 'jour', 'Psychiatr Hung.');) 2005, **20**:463-471. |
| **Mental morbidity** | |
| **daily urgent hospitalisation for mental treatment (except cases of suicidal behaviour)** | Hesdorffer DC, Ishihara L, Mynepalli L, Webb DJ, Weil J, Hauser WA: [**Epilepsy, suicidality, and psychiatric disorders: a bidirectional association.**](http://www.ncbi.nlm.nih.gov/pubmed/22887468) *Ann Neurol* 2012, 72:184-91.  Baxter AJ, Charlson FJ, Somerville AJ, Whiteford HA: [**Mental disorders as risk factors: assessing the evidence for the Global Burden of Disease Study.**](http://www.ncbi.nlm.nih.gov/pubmed/22176705) *BMC Med* 2011, **9**:134. |
| **International, national and city regular events considered as covariates for model building** | |
| **state secular holidays** | [Bozsonyi K](http://www.ncbi.nlm.nih.gov/sites/entrez?Db=pubmed&Cmd=Search&Term="Bozsonyi K"%5BAuthor%5D&itool=EntrezSystem2.PEntrez.Pubmed.Pubmed_ResultsPanel.Pubmed_DiscoveryPanel.Pubmed_RVAbstractPlus), [Veres E](http://www.ncbi.nlm.nih.gov/sites/entrez?Db=pubmed&Cmd=Search&Term="Veres E"%5BAuthor%5D&itool=EntrezSystem2.PEntrez.Pubmed.Pubmed_ResultsPanel.Pubmed_DiscoveryPanel.Pubmed_RVAbstractPlus), [Zonda T](http://www.ncbi.nlm.nih.gov/sites/entrez?Db=pubmed&Cmd=Search&Term="Zonda T"%5BAuthor%5D&itool=EntrezSystem2.PEntrez.Pubmed.Pubmed_ResultsPanel.Pubmed_DiscoveryPanel.Pubmed_RVAbstractPlus): [**The effect of public holidays on the suicide drive (frequency) in Hungary (1970-2002)**]. [*Psychiatr Hung*](javascript:AL_get(this, 'jour', 'Psychiatr Hung.');) 2005, **20**:463-471. |
| **state Orthodox feasts** | Ajdacic-Gross , C . Lauber , M . Bopp , D . Eich , M . Gostynski , F . Gutzwiller , T . Burns , W . Rössler: **Reduction in the suicide rate during Advent—a time series analysis.** *Psychiatry Res*  2008, **157**:139-146. |
| **general elections** | Voracek M, Formann AK, Fülöp G, Sonneck G: [**Suicide and general elections in Austria: do preceding regional suicide rate differentials foreshadow subsequent voting behavior swings?**](http://www.ncbi.nlm.nih.gov/pubmed/12738044) *J Affect Disord* 2003, **74**:257-66. |
